# *COMT* and *ACE* (Epi)genetic Variation Is Associated with Cognitive and Metabolic Resilience in Swiss Tactical Athletes

**DOI:** 10.3390/ijms27031340

**Published:** 2026-01-29

**Authors:** Martin Flück, Christian Protte, Marie-Noëlle Giraud, Eric Häusler, Regula Züger, Alain Dössegger

**Affiliations:** 1 Swiss Federal Institute of Sport Magglingen, SFISM, 2532 Magglingen, Switzerland; alain.doessegger@baspo.admin.ch; 2 Physiogene LLC, Route de Villars 28, 1700 Fribourg, Switzerland; 3 Zentrum für Nieren-Hochdruck- und Stoffwechselerkrankungen, 30625 Hannover, Germany; christian.protte@baspo.admin.ch; 4 Department of Endocrinology, Metabolism and Cardiovascular System (EMC), Faculty of Sciences and Medicine, University of Fribourg, 1700 Fribourg, Switzerland; marie-noelle.giraud@unifr.ch; 5 Percoms AG, Rosenbergstrasse 42, 9000 St. Gallen, Switzerland; eric.haeusler@percoms.ch; 6 Swiss Armed Forces, Armed Forces College, Leadership and Communication Training Command, 6000 Luzern, Switzerland; regula.zueger@vtg.admin.ch

**Keywords:** biomarker, cardiopulmonary, near-infrared spectroscopy, tensiomyography, exercise, soldier, genetic, physical activity, training, performance, cognition

## Abstract

Resilience to stress integrates cognitive, physiological, and behavioral adaptations to sustain performance under adversity. Genetic variation in catechol-O-methyltransferase (*COMT*, rs4680) and angiotensin-converting enzyme (*ACE*, rs1799752) modulates dopaminergic and renin–angiotensin signaling, influencing tissue oxygenation and fatigue resistance. We examined *COMT*- and *ACE*-promoter methylation and genotypes in relation to resilience traits in Swiss tactical athletes (24.6 years) with a maximal power output of 534 W and 21,656 W, respectively, during cardiopulmonary exercise and elbow strike testing. At a 5% false-discovery rate, *COMT* genotype/methylation explained ~12% of the variance in cognitive performance and metabolic resilience, while *ACE* explained ~6–7% in strength-endurance and muscle resistance. Antidromic linear associations between *COMT* genotype and methylation with visual reaction time under reactive stress indicate opposing regulatory influences, best captured by regression models incorporating (epi)genetic covariates. The strongest methylation effects involved *COMT* promoter associations with muscle hemoglobin content across cardiopulmonary exercise zones (r = 0.43–0.58) and sport-specific strain (r = −0.46). *COMT*- and *ACE*-promoter methylation, correlated with time spent in the first aerobic training zone (r = 0.55 and 0.32), indicating environmentally responsive epigenetic modulation. These findings highlight neurovascular–metabolic coupling via dopaminergic and renin–angiotensin pathways as a key mechanism in stress adaptation. System-level adaptations in these pathways align with *COMT* and *ACE* (epi)genetic blood profiles, positioning them as candidate resilience biomarkers. Larger, preregistered studies with site-specific CpG analyses and mechanistic assays are needed to establish causal relevance and translational utility for resilience-informed performance optimization in high-stakes professionals.

## 1. Introduction

Operational effectiveness in military and tactical contexts depends on sustaining cognitive and physical performance under extreme stress, with acute fatigue resistance playing a critical role in preserving executive function [1]. Resilience, defined as the capacity to withstand, adapt to, and recover from adversity without functional decline, is therefore a key determinant of performance in high-intensity military training [2,3,4]. Interindividual stress responses are often conceptualized along a “warrior–worrier” spectrum, reflecting variability in metabolic capacity, stress reactivity, and adaptability [5,6,7].

Catechol-O-methyltransferase (COMT) and angiotensin-converting enzyme (ACE) are central regulators of these processes. COMT degrades catecholamines, thereby modulating dopaminergic tone in the prefrontal cortex and peripheral vasoregulation [8,9,10,11,12]. Dopamine exerts dose-dependent vascular effects, supporting renal, mesenteric, cerebral and skeletal muscle perfusion at low concentrations and inducing vasoconstriction at higher levels [10,11,12,13]. Through these mechanisms, COMT activity links executive control and cognition with neurovascular regulation under stress. ACE regulates cardiovascular and cognitive stress responses by generating angiotensin II, which promotes vasoconstriction and sympathetic–hypothalamic–pituitary–adrenal (HPA) axis activation, and by degrading bradykinin, a mediator of nitric oxide–dependent vasodilation and cerebral perfusion [14,15,16].

Importantly, dopamine and angiotensin II interact at multiple regulatory levels, including vascular control, renal function, and HPA axis activation [11,16,17,18,19]. As a result of this interaction, COMT- and ACE-mediated pathways jointly influence stress tolerance and recovery capacity, as well as cognitive and (psycho)motor performance under sustained load, thereby shaping interindividual differences in functional resilience and mitigating functional decline [17,20,21,22,23,24,25].

Polymorphism rs4680 (*COMT* Val158Met), alters the catalytic efficiency of COMT, with the Met allele conferring approximately 3–4-fold lower enzyme activity than the Val allele [26,27,28]. Likewise, the *ACE* rs1799752 insertion (I) allele is associated with reduced *ACE* expression and enzyme activity relative to the deletion (D) allele [29,30]. These genetic variants therefore establish interindividual differences in baseline dopaminergic and renin–angiotensin signaling within the COMT–ACE stress-regulation axis.

Epigenetic regulation adds further complexity. Methylation of cytosine residues within CpG sites in the promoter region of the genes, including *COMT* and *ACE*, repress transcription and dynamically respond to environmental stressors [31,32,33]. Cytosine methylation within the *ACE*- (−456 to −255) and *COMT*- (−123 to +150) promoter regions in peripheral blood mononuclear cells has been associated with stress exposure, cardiovascular regulation, and cognitive performance [33,34,35,36]. Acute cognitive challenges can induce subtle changes in *COMT*-promoter methylation within one hour [35], while *ACE*-promoter methylation appears sensitive to stressors affecting vascular control [34,36].

Despite extensive pharmacological and genetic evidence, little is known about how combined genetic and epigenetic variation in *COMT* and *ACE* influences fatigue resistance and resilience in real-world, high-stress environments. Here, we examined *COMT*- and *ACE*-genotype and -promoter methylation as indices of gene activity regulation in Swiss tactical athletes, relating them to standardized measures of cognitive control, fatigue resistance, and muscle oxygenation during operationally relevant treadmill protocols. We further assessed associations with training exposure and perceived strain to explore environmentally responsive epigenetic modulation.

## 2. Results

**Figure 1:**
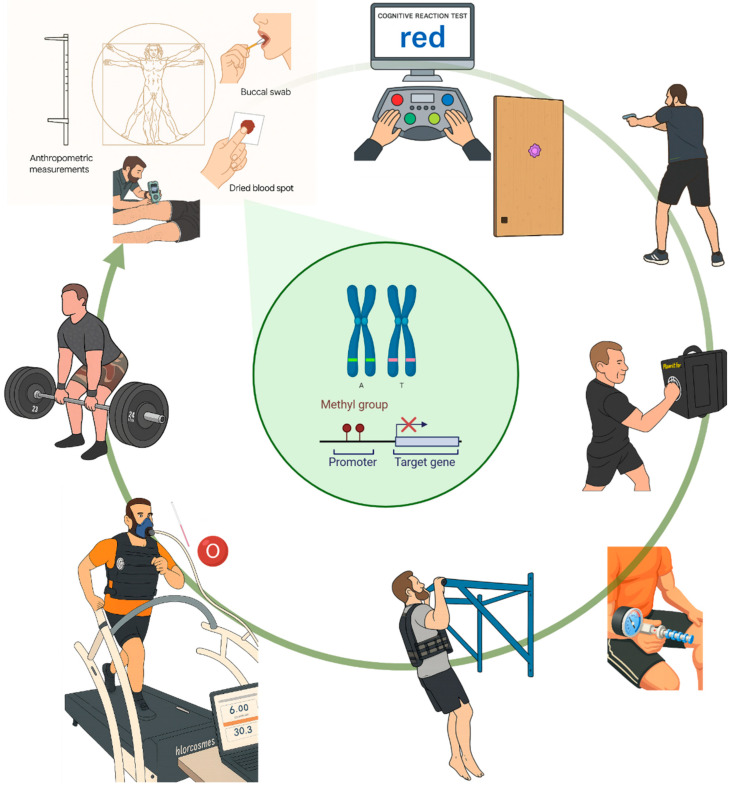
*Overview of the test battery.* Illustration of the alignment of the exercises and sampling that the tactical athletes completed. In sequence, this included anthropometry, the completion of a questionnaire on training behavior, and training intentions, a STROOP and determination test (DT) to assess resistance of cognitive performance, a shoot and move test to assess reaction times to visual stimuli, a test for leg and arm impact power, handgrip strength, pullups with a weight vest to assess upper body strength endurance, uphill running to exhaustion on a treadmill with a weight vest to assess fatigue resistance and systemic and muscle parameters of aerobic/anaerobic metabolism, and all-out deadlift-exercise. This was accompanied by measures for acute mechanical resistance of muscle by tensiomyography, and collection of mucosa and blood spots to assess the genotype and promotor methylation for the *ACE* and *COMT* genes (illustrated in the center). Created in BioRender. Dössegger, A. (2026) https://BioRender.com/phr8h0r (accessed on 25 September 2025).

To test whether the *COMT*- and *ACE*-genotype and -promoter methylation relate to proxies of resilience under operational stress, we first characterized the physiological and cognitive performance of Swiss tactical athletes (Figure 1), and then examined how molecular variation aligned with systemic, muscular, and behavioral indices across the integrated test battery.

### 2.1. Subject Characteristics

Table 1 summarizes the anthropometric, cognitive, and physical performance characteristics, as well as training behavior and perceived strain, of the 61 male Swiss tactical athletes studied.

Compared to reference values from active populations, participants demonstrated good-to-very-good levels of strength, strength–endurance, and aerobic/anaerobic capacity [37,38,39,40] under exercise tests with additional load to simulate the operational demand of tactical athletes. Specifically, body-mass-related power outputs reached values of 6.6 ± 0.5 [5.5–7.5] Watt kg^−1^ and 6.8 ± 0.5 [5.7–8.0] Watt kg^−1^, for the body-mass-related power output at maximal oxygen uptake (P_VO_2_max), and the maximal power output on the treadmill (maxP_end).

Cognitive performance was overall average relative to normative samples [41], with individual variability in the response time under reactive stress conditions (i.e., DT_rt and DT_pr_rt, respectively), and cognitive performance to incongruent stimuli in the color–word Stroop test(i.e., STROOP_pr_R and STROOP_pr_W), consistent with a normal distribution.

### 2.2. Metabolic and Mechanical Aspects of Resilience

**Figure 2:**
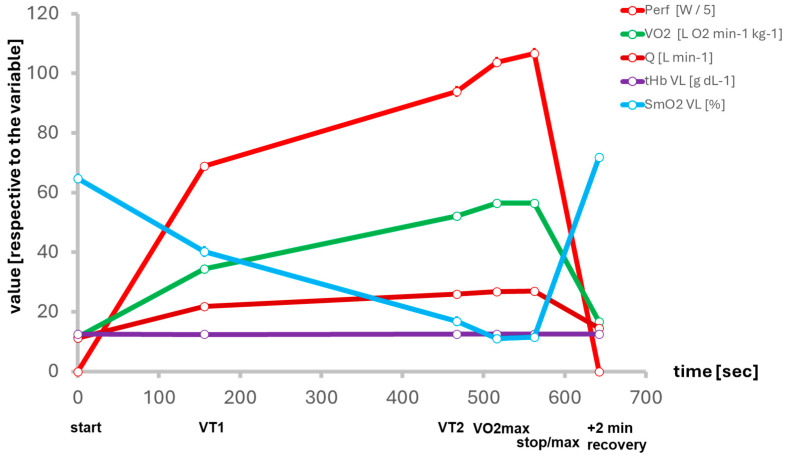
*Characteristics of fatigue resistance to uphill running until exhaustion.* Line graph of the mean ± standard error for cardio-respiratory parameters at respective time points for a ventilatory threshold during the conducted running exercise until exhaustion with a loaded vest. Abbreviations: Perf, performance; VO_2_, oxygen uptake; Q, cardiac output; tHb VL, hemoglobin content in vastus lateralis muscle; SmO_2_ VL, oxygen saturation in vastus lateralis muscle. *N* = 61.

During loaded uphill cardiopulmonary exercise testing (CPET), systemic and local indices of fatigue resilience showed pronounced perturbations at the systemic and local muscle levels (Figure 2; Table S2). Oxygen saturation (SmO_2_) in the m. vastus lateralis and m. gastrocnemius decreased four-fold, reaching minima of 11.7% and 13.8%, respectively. Peak oxygen uptake averaged 56.7 ± 6.0 [43.0–72.0] mL O_2_ min^−1^ kg^−1^, with exhaustion occurring after ~563 s at an incline of 8.8°. Serum lactate rose sharply to 13.5 ± 3.0 mmol·L^−1^ [range: 5.4–21.0] 5 min post-exercise, underscoring reliance on anaerobic metabolism in the final exercise phase. Cardiac output doubled relative to rest (26.9 L·min^−1^), while total hemoglobin concentration (tHb) in m. vastus lateralis remained stable, indicating preserved perfusion (Table S2).

Mechanical fatigue was reflected in increased stiffness and tone of *m. vastus lateralis*, whereas other muscles were unaffected. Recovery kinetics diverged: local muscle oxygenation fully normalized within 2 min (m. vastus lateralis 113.8%, *m. gastrocnemius* 106.8% of baseline values), while systemic markers recovered only partially (cardiac output 78.2%, oxygen uptake 88.0%, and glucose at 57.8% of baseline values).

Systemic oxygen uptake and muscle oxygen saturation (SmO_2_) in the m. vastus lateralis and gastrocnemius exhibited reductions of approximately 80%during the ramp CPET. Minimal values of 11.7% and 13.6% for oxygen saturation in the vastus lateralis and gastrocnemius muscles were measured. Maximal oxygen uptake (VO_2_max) was 56.7 ± 6.0 [43.0–72.0] mL O_2_ min^−1^ kg^−1^ when subjects terminated running at an average incline of 8.8° after an average of 563 s running time.

Metabolic strain—also being reflective of metabolic capacity—was further evidenced by a marked increase in serum lactate, peaking at 13.5 ± 3.0 mmol·L^−1^ [range: 5.4–21.0] within 5 min post-exercise, underscoring the heavy reliance on anaerobic metabolism in the final exercise phase. Cardiac output reached an average of 26.9 L·min^−1^, corresponding to an estimated two-fold increase in cardiac workload relative to resting conditions. Total hemoglobin concentration (tHb) in the vastus lateralis remained stable, suggesting that local perfusion was preserved during exercise (Table S2).

In terms of mechanical resistance, loaded uphill running to exhaustion induced changes in muscle stiffness and tone, specifically within the vastus lateralis, while no significant effects were noted for other recruited muscles. These alterations are indicative of local mechanical fatigue. During the two-minute post-exercise recovery, SmO_2_ in the m. vastus lateralis demonstrated complete restoration to the baseline value, where SmO_2_ recovered in the m. vastus lateralis to 113.8%, and in the m. gastrocnemius to 106.8% of the values at baseline. In contrast, the systemic markers, of cardiac output (Q), VO_2_, and blood glucose recovered to 78.2%, 88.0%, and 57.8% of baseline values, respectively.

### 2.3. Genetic and Epigenetic Characteristics

For the *ACE* gene polymorphism rs1799752, genotype frequencies showed an excess of heterozygotes relative to Hardy–Weinberg expectations (observed: 38 *ID*, 11 *II*, 11 *DD*; expected *ID* ≈ 30). The chi-square test indicated a significant deviation from equilibrium (χ^2^(1) = 4.27, *p* = 0.039), whereas the exact test yielded a borderline result (*p* = 0.069). Bayesian analysis favored the saturated model (BF_10_ = 0.44; Jeffreys priors: BF_10_ = 0.51), providing weak-to-moderate evidence against HWE.

Genotype frequencies for the *COMT* gene polymorphism rs4680 (GG = 15, GA = 29, and AA = 16) were consistent with the Hardy–Weinberg equilibrium. Both the chi-square test (χ^2^(1) = 0.07, *p* = 0.80) and the exact test (*p* = 0.80) supported the equilibrium. Bayesian analysis also favored the HWE model (BF_10_ = 3.04; Jeffreys priors: BF_10_ = 4.28), indicating moderate evidence for equilibrium.

**Figure 3:**
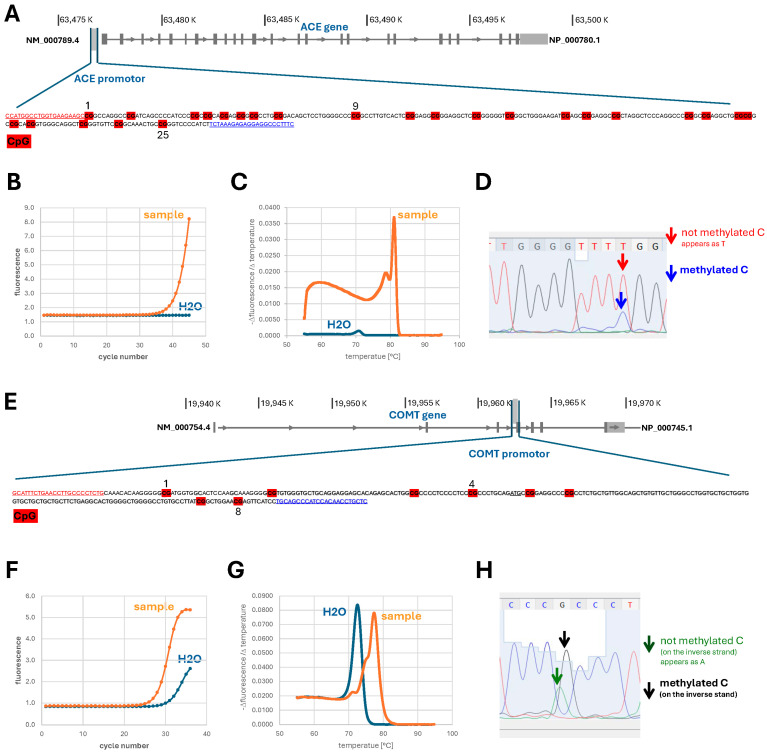
*Assessment of ACE and COMT promoter methylation.* (**A**,**E**) Structure of the *ACE* (**A**) and *COMT* (**E**) gene with the enlarged promoter regions, from positions −456 to −255 and −123 to 150, respectively, that were assessed for the methylation of contained 25, or 8, CpG dinucleotides (highlighted in red). The sequences in underlined, red and blue font correspond to the positions of the forward and reverse primers, without considering cytosine methylation and bisulfite induced methylation. In the *COMT* gene sequence, the initiation site for protein translation is underlined. (**B**,**C**,**F**,**G**) Examples of the fluorescence-based detection of the PCR-based amplification (**B**,**F**) and melting curve (**C**,**G**) of the respective *ACE*- (**A**,**C**) and *COMT*- (**F**,**G**) promoter regions after BIS-conversion in a sample compared to a non-template control (H2O). (**D**,**H**) Annotated part of the chromatogram from Sanger-based sequencing of the *ACE* (**D**) and *COMT* promoter (**H**), respectively. For the *ACE* promoter, the chromatogram depicts the sequence around CpG dinucleotide no 9, where red and blue arrows, respectively, point to an un-methylated and methylated cytosine (C) residue that is converted by the bisulfite reaction into a thymidine (T), or is protected due tomethylation. For the *COMT* promoter the chromatogram depicts the sequence around the CpG dinucleotide no. 4, whereby green and black arrows, respectively, point to the un-methylated and methylated cytosine that is converted into thymidine on the inverse sequence, or is protected due to methylation.

**Figure 4:**
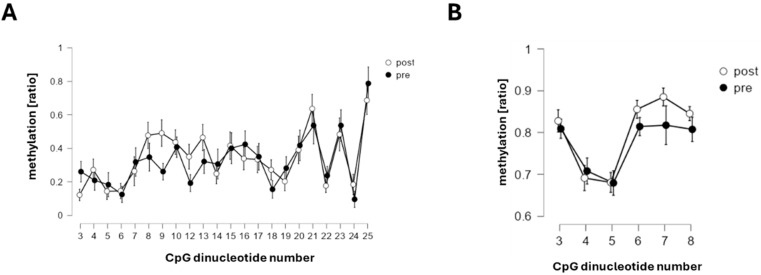
*CpG methylation patterns in ACE and COMT promoters*. Line graph with the standard error of the ratio of methylation for the reliably detected CpG dinucleotides in the *ACE* (**A**) and *COMT* (**B**) promoter for the 29 cases where samples were available prior to and after the test battery.

Reliable methylation was detected at 6/8 CpGs in the *COMT* promoter and 22/25 CpGs in the *ACE* promoter, with distinct site-specific methylation patterns for both promoters (*p* < 0.001; *COMT*: CpGs 1, 6–8; *ACE*: CpGs 21, 23, 25; see Figure 3 and Figure 4).

### 2.4. Association of ACE- and COMT-Promoter Methylation and Genotype with Performance and Aspects of Resilience

**Figure 5:**
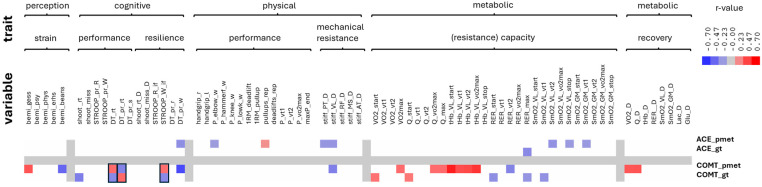
*Correlations between (epi)genetic variations and traits of physical and cognitive resilience.* Heatmap visualizing significant positive (red) and negative (blue) linear relationships between variables that contribute to six traits of resilience (i.e., perceived strain, cognitive resilience, physical strength, mechanical resistance, metabolic capacity, metabolic recovery) and promoter methylation and genotypes for the *ACE* and *COMT* genes, i.e., ACE_pmet and ACE_gt vs. COMT_pmet and COMT_gt. Calculations were based on Pearson’s moment correlations and displayed using cluster software. *p*-values were adjusted for false-discovery-rates at a threshold of 5%. Boxed areas indicate parameters for which antidromic relationships were identified both for the ratio of promoter methylation and genotype. Note the specific pattern of relationships, which emphasizes the association of the *ACE* gene with parameters characterizing mechanical and metabolic resistance of skeletal muscle, and the *COMT* gene with cognitive performance, cardiovascular and muscular aspects of metabolism. *N* = 61. For further explanation we refer to the list of abbreviations and Table S1.

Across 108 variables, 933 significant correlations (FDR-adjusted *p* < 0.05) were identified, including 54 involving the *ACE/COMT* genotype or promoter methylation (effect sizes: r = −0.592 to 0.580; Figure 5). Figure S1 (also see Table S1) depicts aspects of the resulting network of linear associations between the investigated genetic and epigenetic features and variables reflecting cognitive and physical resilience and metabolic resistance, highlighting interconnections among these functional domains that are mediated by (epi)genetic regulation of *ACE* and *COMT* gene activity.

Collectively, these explained ~11% of the variance in resilience-related outcomes.

*COMT*-promoter methylation correlated positively with hemoglobin content in *m. vastus lateralis* at baseline and across exercise intensities (*r* = 0.43–0.58) and maximal oxygen uptake (*r* = 0.33), and negatively with the percentage rank of incorrect responses in the determination test (DT_pr_w; *r* = −0.59). Additional associations included Stroop interference (STROOP_W_if, *r* = 0.32), reaction time (DT_rt, *r* = 0.33), cardiac output (*r* = 0.33–0.41), and glucose regulation before and after CPET testing (*r* = 0.35–0.41).

*COMT* genotype (G-allele copy number) correlated negatively with shooting reaction time (*r* = −0.36) and respiratory exchange ratio at the start and maxima (*r* = −0.28 and −0.26, respectively), but positively with oxygen uptake and cardiac output at exercise onset (*r* = 0.29–0.31, respectively).

*ACE*-promoter methylation was linked to endurance-related traits, including loaded pull-ups (*r* = 0.21), muscle oxygenation indices at varying intensities/ventilatory thresholds of endurance exercise (*r* = −0.23 to −0.27), *m. plantaris* stiffness (stiff_PT_D, *r* = −0.25), and elbow striking power (*r* = −0.22). It also correlated with post-exercise lactate (*r* = 0.23).

No significant correlation was found between *ACE*- and *COMT*-promoter methylation (*p* = 0.39). Both genotypes correlated negatively with the percentage of wrong answers in the determination test (DT_pr_w; r = −0.28 and r = −0.59, respectively) and mechanical stiffness of m. vastus lateralis (stiff_VL_D; r = −0.26 and r = −0.35). *ACE* and *COMT* genotype were borderline unrelated (*p* = 0.058), when correlations emerged for both versus the reflector of the metabolic effort during uphill running, (RER_max; r = −0.31 and r = −0.26).

Antidromic associations emerged for *COMT* genotype and methylation with the determination test reaction time (DT_rt), its percentile rank (DT_pr_rt), and word interference in the Stroop test (STROOP_W_if), indicating opposing regulatory effects.

### 2.5. Relationship Between COMT and ACE Gene Promoter Methylation with Physical Activity and Experienced Strain

**Figure 6:**
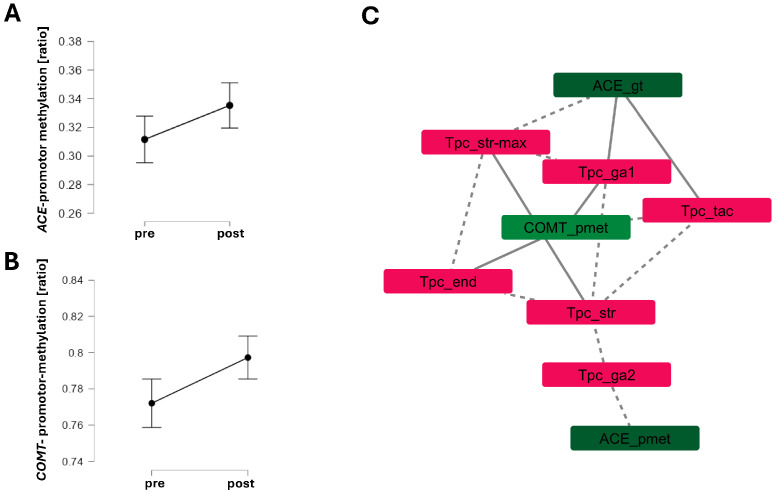
*Links between promoter methylation and training behavior.* (**A**,**B**) Line graph with the standard error of the average ratio of CpG methylation for the *ACE* (**A**) and *COMT* (**B**) promoter per individual CpG dinucleotide, i.e., ACE-_pmet and COMT-_pmet (**A**,**B**). (**C**) Network of linear relationships between *ACE* and *COMT* promoter methylation and genotype with the levels of physical activity assessed in the week before the test battery. Positive and negative linear relationships passing a false discovery-rate-adjusted threshold of 5% are indicated by connected and stippled lines, respectively. Low-and high-intensity zones of aerobic exercise are labeled as ga1 and ga2—the first- and second-zone training of basic endurance (Ger.: Grundlagen–Ausdauer). Only first-order relationships with indices of physical activity are shown. *N* = 61.

In the subset with pre- and post-test methylation data (*n* = 29), average *COMT*-promoter methylation was three-fold higher than *ACE* methylation, with no significant pre–post differences (Figure 6).

*COMT* promoter methylation correlated with time in the first aerobic zone (*r* = 0.55), endurance training volume (*r* = 0.44), and overall perceived strain/recovery (bemi_gess; *r* = 0.41), but inversely with sport-specific strain (bemi_beans: *r* = −0.46) and tactical training time (*r* = −0.37). *ACE* promoter methylation correlated weakly and negatively with time in the second aerobic zone (*r* = −0.22), while *ACE* genotype associated positively with aerobic zone 1 training (*r* = 0.32), tactical training time (*r* = 0.23), and negatively with maximal strength training (*r* = −0.33).

The levels of (the average) *COMT* (*p* = 0.564,) and *ACE* promoter (*p* = 0.92, η^2^ < 0.001) methylation did not differ between before and after the test battery of cognitive and physical exercise, and this was not essentially affected when the CpG dinucleotide number was considered (*COMT*: *p* = 0.12; *ACE*: *p* = 0.42).

### 2.6. Regression Analysis

**Figure 7:**
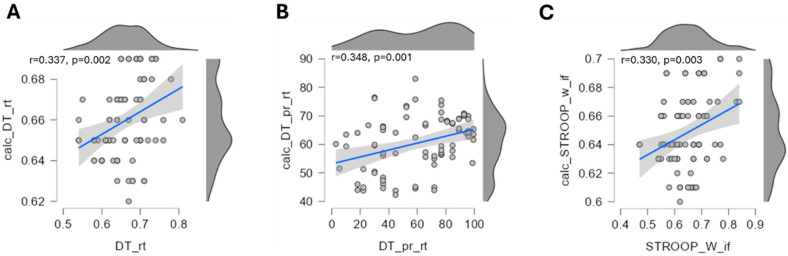
*In-sample fit of (epi)genetic influences.* Scatter plots with the regression lines and 95% confidence intervals for three variables of cognitive performance and resilience, i.e., DT_rt (**A**), DT_pr_rt (**B**), STROOP_W_if (**C**), for which a correlative association with *ACE/COMT* genotypes and promoter methylation was detected based on regression analysis (*p* < 0.01) and a calculated correlation based on the resulting regression model (*p* < 0.01). X-axes represent the effectively measured and Y-axes the calculated values as models based on a multi-correlation of the four variables *ACE* genotype, *ACE* promoter methylation, *COMT* genotype and *COMT* promoter methylation. *R*-values and *p*-values for linear relationships between measured and calculated values are shown in each scatter plot. *N* = 61.

Eight resilience-related variables showed significant associations with *ACE/COMT* (epi)genetic markers (*p* < 0.01). Cognitive variables—such as the determination test reaction time and its percentile rank, and Stroop writing interference—demonstrated modest but significant model fits (variance-adjusted *r*^2^ = 0.10–0.13, *p* < 0.005; Figure 7), with the *COMT* methylation and genotype explaining ~12% of the variance. No significant regression models were observed for physical or metabolic/mechanical variables of resistance (Figure S2).

## 3. Discussion

Resilience—the capacity to sustain psychological and physical performance under stress—, and stress resistance is increasingly recognized as a multisystem construct critical for operational effectiveness in tactical athletes [2,3,4]. It encompasses (amongst other elements) cardiovascular and metabolic buffering, cognitive control, neuroendocrine modulation, and adaptive behavior under stress, with key contributions from the HPA axis and physical activity [42,43,44,45,46]. Our study operationalized this multidimensionality through a comprehensive test battery in a real-world tactical setting (Figure 1), spanning systemic and muscular responses to exertion (Figure 2), (epi)genetic regulation of *COMT* and *ACE* (Figure 3 and Figure 4), trait associations (Figure 5), training exposure (Figure 6), and an epigenetic model of cognitive performance (Figure 7).

*COMT*- and *ACE*-genotype and -promoter methylation showed significant associations with partially distinct resilience domains. *COMT*-related markers were most strongly associated with cognitive performance under stress and training behavior/strain, whereas *ACE*-related markers were more closely associated with endurance- and mechanical-resistance traits and striking power, with minimal association with cognitive metrics. Across models, cognitive outcomes showed modest but consistent fit (adjusted *r*^2^ ≈ 0.10–0.13), whereas physical and metabolic/mechanical outcomes showed weaker predictive structure. This pattern supports complementary contributions of *COMT*- and *ACE*-related regulation to cognitive and physical robustness and resilience phenotypes in tactical athletes.

Taken together, the results identify a coherent association pattern linking *COMT*- and *ACE*-related (epi)genetic variation with (i) cognitive performance under stress, (ii) systemic and local oxygen delivery, and (iii) endurance-related mechanical and metabolic traits. The following sections interpret this pattern from complementary physiological and regulatory perspectives. Because these data are correlational and based on whole-blood methylation, the interpretations below emphasize plausible regulatory links rather than causal mechanisms.

### 3.1. Epigenetic Lens: Training Load and Environmentally Responsive Methylation

Although *ACE* and *COMT* promoter methylation remained stable post-exercise (Figure 4), their linear associations with weekly training volume and perceived strain suggest that they reflect habitual training load, consistent with environmentally responsive CpG methylation. Prior studies have shown that acute stressors, such as transcranial stimulation, can induce subtle changes in *COMT* promoter methylation (~0.5%) [35]. In our cohort, *COMT* and *ACE* methylation levels correlated positively and negatively, respectively, with time spent in aerobic intensity zones 1 and 2; this is in line with the evidence that physical activity induces tissue-wide methylation changes [47]. Therefore, the directionality of the linear relationship thereby was consistent with peripheral blood studies, reporting associations between physical activity and *COMT* promoter methylation [48], and reduced *ACE* promoter methylation following combined cardiovascular and strength training [49]. Given the weekly turnover of white blood cell–derived DNA [50], observed methylation patterns likely capture cumulative responses to repeated stressors.

Three antidromic relationships were identified between *COMT*-genotype and promoter methylation. Although modeled linearly, these patterns may reflect nonlinear feedback within stress-regulatory circuitry [51,52]. *COMT*- and *ACE*-promoter methylation were linked to time spent in aerobic zones 1 and 2, respectively—zones typically used for long aerobic effort, recovery, or enhancing aerobic power [53]. *COMT*-promoter methylation rose in zone 1, suggesting diminished gene activity, whereas *ACE*-promoter methylation declined in zone 2, consistent with enhanced *ACE* expression [54,55]. The *ACE* I/D genotype further correlated with aerobic zone 1 training and maximal strength training, aligning with prior findings on energy expenditure and endurance capacity [56,57]. This genotype also showed a moderate association with maximal respiration rates (r = −0.311), consistent with *ACE* I/D–related differences in muscle aerobic metabolism during exhaustive endurance exercise in healthy male subjects [30].

### 3.2. Neurocognitive Lens: COMT-Related Associations with Executive Control Under Stress

*COMT*-genotype and -promoter methylation were individually associated with cognitive performance metrics, but in opposing directions. Lower *COMT*-promoter methylation corresponded to slower reaction times in the determination test and diminished proactive control in the Stroop test, yet these variables demonstrated the best in-sample model fit when combined in regression (Figure 7A,B). These findings align with prior reports linking *COMT* rs4680 to cognition under stress [58,59,60], and suggest that reduced *COMT* methylation may confer selective advantages in cognitive flexibility under high strain.

### 3.3. Neurovascular–Metabolic Lens: Oxygen Delivery and Substrate Use During Loaded Running

*COMT* genotype and methylation also showed opposing correlations with cardiorespiratory variables during loaded uphill running (VO_2_max, Qmax, and tHb_VL_vo2max), consistent with a dopaminergic mechanism supporting both cognitive and physical adaptability [61,62]. Similarly, *ACE*-promoter methylation correlated negatively with oxygenation of vastus lateralis and gastrocnemius medialis during running, consistent with prior evidence that lower ACE activity (pharmacological inhibition or I-allele carriage) is associated with improved muscle perfusion [63,64,65]. The *ACE* I/D polymorphism was also shown to be associated with RER_max, echoing prior findings on *ACE*-related differences in aerobic metabolism [30,56,57,64,66].

### 3.4. Biomechanical Lens: Stiffness and Mechanical Fatigue Resistance

Exercise-induced changes in muscle stiffness correlated with *ACE*-promoter methylation, suggesting biomechanical modulation of fatigue resistance by *ACE* gene activity [67,68]. Parallel associations between *COMT*-promoter methylation and indices of muscle perfusion (tHb, SmO_2_, and RER) further support a role for *COMT* gene activity in cardiovascular–metabolic coupling (Figure S1; [26,69,70]). The consistency of the tHb associations across intensities supports a stable link between COMT-related regulation and perfusion indices during exertion; relating to dopamine-stimulated vasoreactivity in skeletal muscle. These findings mirror reports of increased forebrain and muscle perfusion in *COMT* rs4680 G-allele carriers under stress [60,71].

### 3.5. Metabolic Lens: Glucose Flux, Catecholamines, and Methylation

Promoter methylation of CpG sites in blood and adipose tissue has been linked to dietary glycemic load, particularly in maternal nutrition and offspring epigenetic programming [72,73]. Elevated glucose concentrations can also alter DNA methylation in human neuronal progenitor cells, affecting neurodevelopmental pathways such as SLIT1–ROBO2 and Hippo signaling [74]. In this context, the observed correlation between COMT-promoter methylation, post-exercise RER at ventilatory threshold 2, and increased blood glucose concentration following exhaustive uphill running is notable. This association is consistent with catecholamine-driven glucose flux during intense exercise, a process tightly regulated by catecholamines degraded by COMT [75,76]. Similarly, the linear relationship between vastus lateralis oxygenation at ventilatory threshold 2 and *ACE*-promoter methylation provides mechanistic insight, echoing reports of the *ACE* I/D genotype-dependent override of angiotensin II-mediated vasoconstriction [65]. Importantly, *ACE*-promoter methylation was unrelated to blood glucose (*p* = 0.887), suggesting a distinct regulatory axis. Collectively, these findings raise the possibility that *COMT*-promoter methylation reflects a causal response to glucose flux and catecholamine turnover rather than a downstream effect of hyperglycemia, warranting further study of repeated exercise-induced glucose fluctuations and metabolic stress in shaping DNA methylation profiles.

### 3.6. Translational Lens: From Clinical Genetics to High-Performing Tactical Cohorts

Together, these results suggest that *COMT* and *ACE* (epi)genetic regulation extends beyond clinical contexts (e.g., depression, schizophrenia, and trauma) [34,36,54,77,78,79] to healthy, high-performing populations. Both genes regulate the HPA axis [11,17,19] with dopamine and angiotensin II contributing to individual differences in resilience across cognitive and physical domains. Our findings support the view that genotypes typically linked to borderline cognitive traits may confer performance advantages under stress, particularly through epigenetic regulation of *COMT*.

### 3.7. Integrative Lens: Brain–Muscle Coupling as a Shared Substrate of Resilience

The observed associations between muscle perfusion, oxygenation, and cognitive performance highlight aerobic muscle metabolism as a shared substrate for neuromuscular and neurocognitive resilience. This aligns with evidence that endurance exercise induces mitochondrial adaptations that enhance fatigue resistance and cognitive function [80]. This integrative interpretation complements the training–*COMT*-promoter methylation associations described above (see Figure 6), suggesting a potential conditioning of brain–muscle coupling by repeated endurance exposure.

From a pathway-interaction perspective, a subset of shared associations points to potential coupling between dopaminergic and angiotensin signaling. Negative correlations between *ACE*- and *COMT*-gene promoter methylation and the percentage rank of wrong answers in the determination test (DT_pr_w) suggest interactions between angiotensin and dopaminergic pathways, consistent with angiotensin’s modulation of dopamine release [17,81]. Elevated ACE activity, expected in DD genotypes or low promoter methylation [31,82], has been linked to reduced processing speed and working memory in aging [83]. In our data, *ACE* and *COMT* associations co-occurred in RER and muscle stiffness with physical exertion, suggesting combined influence under high energy demand in the tactical athletes studied.

While tissue-level mechanisms remain speculative, linear relationships of *COMT*- and *ACE*-promoter methylation with VO_2_, cardiac output, hemoglobin content, and maximal RER support their role in systemic oxygen delivery during exertion [71]. These effects are likely mediated by dopamine and angiotensin signaling on vascular and muscle tissues [14,64]. The association of the *COMT* rs4680 GG genotype with increased forebrain oxygenation during cognitive demand [60] reinforces the plausibility of a neurovascular mechanism underlying resilience and the distinction of “warrior”/“worrier” phenotypes.

### 3.8. Limitations

This exploratory study is limited by a modest sample size, the absence of direct enzyme-activity measures, and reliance on whole-blood methylation, which reflects circulating leukocytes with short turnover [50]. Our analysis also focused on mean promoter methylation rather than specific CpG sites due to limited knowledge about the exact positions and transcription factor binding motifs that regulate *COMT* and *ACE* gene activity in relation to cognitive and physical resilience. Potential methylation-sensitive transcription factors interacting with GC-rich motifs in these promoters include Sp1, AP-2, KLF4, and CTCF [84,85]. Future studies should investigate exercise-induced regulation of specific CpG sites—particularly positions 123–150 in the *COMT* promoter and −456 to −255 in the *ACE* promoter—based on observed associations with training intensity zones and perceived strain. Our data cannot specify the tissue-level molecular processes underlying the observed correlations, as *ACE* and *COMT* enzymes are systemically distributed, and we did not quantify the respective enzymatic activities [86,87]. Environmental exposures such as diet, sleep, and alcohol use may have contributed to methylation variability [88,89,90] beyond the identified associations with exercise behavior and perceived strain. Further aspects of the experimental setup and test sequence may have influenced specific associations—particularly those involving assessments sensitive to warm-up effects and intensity-dependent metabolic activation, such as angiotensin II-mediated vasoconstriction [65]. In addition, although we note that the cognitive performance of the tactical athlete cohort may resemble that of the general population, their physical performance metrics and *ACE I/D* genotype distribution differ from data reported in non-athletic white Caucasian populations with a similar ethnic background while using the same methodology [91,92], potentially affecting physiological outcomes. Intriguingly, the observed excess of *ACE I/D* heterozygotes resembles the over-dominant influence of this genotype on contractile parameters of cardiac and skeletal muscle function in sedentary Swiss populations, which are opposingly affected by the *ACE I* and D allele (reviewed in [91,93,94]).

In the present cohort of Swiss tactical athletes, the *ACE* rs1799752 polymorphism showed a consistent excess of heterozygotes, reflected by a borderline exact test result and weak-to-moderate Bayesian evidence against the Hardy–Weinberg equilibrium (HWE). Several considerations are essential for interpreting this finding. First, a technical artefact is unlikely. Genotyping followed validated protocols, and all ambiguous calls were resolved through repeated reactions and confirmatory Sanger sequencing by an independent commercial laboratory. This substantially reduces the probability that the observed heterozygote excess results from assay errors. Second, deviation from the HWE does not inherently indicate poor data quality in a selected cohort. The HWE is a population-level expectation under random mating and in the absence of selection. Tactical athletes constitute a highly non-random, self-selected, and performance-screened subset of the population. In such groups, departures from the HWE—particularly at loci with known physiological relevance such as rs1799752 [64,66,82,95]—may plausibly arise from selection processes rather than methodological bias. Third, the pattern of deviation is biologically structured rather than stochastic. The excess of *ID* heterozygotes at the *ACE* locus aligns with hypotheses of a heterozygote advantage or selection for mixed endurance–strength phenotypes [95,96], which are central to tactical occupational demands. Although the present study cannot establish causality, the directionality of the deviation argues against random fluctuation alone. Fourth, the Bayesian analysis supports a cautious, graded interpretation. In line with Wakefield’s framework, the Bayes factor provides only weak-to-moderate evidence against the HWE, suggesting that the deviation should be viewed as a potentially meaningful signal rather than a definitive violation. Such nuance is particularly appropriate in modestly sized, highly specialized cohorts where strict adherence to population-genetic null models is unlikely.

In this study, we characterized participants using a test battery specifically developed to capture the phenotypic profile of tactical athletes, including cognitive attributes, aerobic and power-related capacities within a multidimensional fitness framework [97]. To assess cardiopulmonary performance under conditions relevant to the weight-bearing operational demands of this population, we employed a treadmill running protocol with a loaded vest, which reflects the fatigue-resistant load-carrying tasks typical for these athletes [98]. Consequently, the interpretation of cardiorespiratory parameters and their associations with *ACE/COMT* (epi)genotypes may only be comparable to a limited or uncertain extent with indices derived from different types of cardiopulmonary exercise testing including contemporary indices associated with aerobic metabolic capacity in highly trained endurance athletes [99]. This distinction is important, as our protocol was intentionally designed to leverage the strength-based phenotype we recently documented in this population. Moreover, differences in muscle recruitment patterns between different leg exercise modalities—particularly in muscles such as the gastrocnemius and vastus lateralis—may further influence the interpretation of genotype–phenotype associations. This is relevant given our previous findings of *ACE I/D* genotype-dependent plasticity in mitochondrial volume density in response to running and leg cycling type endurance training, which underpins local aerobic capacity [91]. The regulatory mechanisms underlying these interactions remain insufficiently understood and warrant further investigation.

Aforementioned methodological and biological factors likely contributed to variability in the strength and reliability of the observed associations. In the high-dimensional setting of our investigation, modest effect sizes are expected because physiological and cognitive traits are multifactorial, with variance distributed across many interrelated predictors rather than dominated by a single variable. Within this framework, even small correlations can be meaningful when they appear consistently across analytical approaches, as seen in our data (Figure 5).

Across two devices assessing systemic and local markers of aerobic metabolism (VO_2_, Q, and SmO_2_) during the test modality of uphill running to exhaustion, associations of *COMT* genotype replicated directionally, at exercise onset and at the first ventilatory threshold, which supports robustness despite modest effect sizes. *COMT*-promoter methylation likewise showed reproducible relationships with metabolic indices of central and peripheral cardiovascular function (Q and tHb in the *vastus lateralis*) across a broad range of intensities, including maximal workloads. Similar consistency characterized the associations between *ACE*-promoter methylation and SmO_2_ in both the *vastus lateralis* and *gastrocnemius medialis*. Linear relationships involving the *COMT* genotype and *COMT*-promoter methylation with cognitive-resilience indices (DT_rt and STROOP_W_if), assessed using different tests and paradigms, further support the robustness of these findings despite their modest magnitude. Small correlations may still hold theoretical relevance when aligned with established physiological mechanisms or prior evidence. In our dataset, this is reflected by directionally consistent patterns that together form a coherent interpretation, even when individual coefficients are small. This is particularly pertinent given the extensive evidence implicating dopaminergic and angiotensin pathways in physical and cognitive functioning and adaptability [8,9,10,13,14,15,19,60,64,66,69,70,83,93,100,101].

To mitigate the risk of false positives inherent in high-dimensional testing, we applied false discovery rate correction for multiple comparisons and conducted a retrospective power analysis. The most statistically significant Pearson correlations were of moderate strength and supported by power estimates ≥ 0.80. A smaller subset of associations exhibited lower power (0.63–0.80; Table S3), and these should therefore be interpreted with appropriate caution.

### 3.9. Perspectives

Despite these limitations, our exploratory study identified biologically plausible associations between *COMT* and *ACE* (epi)genetic variation and resilience-related traits through a comprehensive functional assessment, integrating targeted cognitive and physical testing with advanced sensor technologies (e.g., near-infrared spectroscopy and tensiomyography) to probe metabolic and mechanical resilience. The observed associations align with prior pharmacological and genetic evidence of the contribution of *ACE* and *COMT* to (psycho)motor and cognitive performance and enhancing fatigue resistance with exhaustive exercise [22,23,24,25,102,103,104]. These findings highlight neurovascular–metabolic coupling as a substrate of stress adaptation and nominate *COMT* and *ACE* as candidate biomarkers for resilience-informed performance optimization. Future work should employ larger, preregistered cohorts, site-specific CpG analyses, and mechanistic assays to establish causal relevance and translational utility.

## 4. Materials and Methods

### 4.1. Ethics

This study adhered to the Declaration of Helsinki (2013 revision) and received approval from the Human Research Ethics Committee of the Canton of Berne (approval no. 2022-00767), and the research committee of the Swiss Armed Forces (Forschungsausschuss San D Armee, Kompetenzentrum Militär und Katastrophenmedizin, 2022-00767), approval date 10 May 2022.

### 4.2. Subjects

Sixty-one male white Caucasians, five operators, and fifty-six candidates for special operation forces were recruited from Swiss military and police personnel, spanning professional and militia units across multiple cantons and formations (e.g., Military Police SOF, Grenadiers, and Parachute Reconnaissance). They were part of a larger cohort of subjects, as described elsewhere [97]. Recruitment was coordinated through unit commands, who distributed study information and consent documents. Participation was voluntary, with confidentiality assured and no impact on recruitment outcomes. Written informed consent was obtained, and each participant was anonymized via a unique study identifier.

Inclusion criteria required all participants to be free from injury, illness, medication affecting heart rate, chronic diseases, and Long COVID—as assessed by a pre-participation examination questionnaire (adapted from the Sports Medicine Pre-participation Examination Questionnaire (v01.02.2023) of the Society Sport and Exercise Medicine Switzerland (SEMS))—and to meet basic non-physical/cognitive standards set by recruiting authorities.

### 4.3. Study Design

Subjects completed a battery of exercises at an arsenal in Switzerland to assess cognitive and physical performance under different stressors, yielding an integrated profile of metabolic and mechanical function and their resistance across a selected set of measurements. Figure 1 visualizes the setup of the fixed-ordered cognitive assessments and fitness tests that were carried out, as described elsewhere [71,97], and Table S1 describes the assessed metabolic and mechanical response variables that characterize aspects of resilience.

The test sequence included cognitive performance using the Vienna Test System (Testform 7, Schuhfried GmbH, Moedling, Austria), assessed by the Stroop resistance of cognitive performance to incongruent stimuli in a color–word interference tendency test [41]. The Stroop test was utilized to assess speed performance in reading words and naming colors, as well as to assess speed performance under color–word interference conditions. Further, the determination test (DT) for the reactive stress tolerance and reaction speed [105] was performed and used to calculate percentile ranks with respect to a representative normative sample, which was stratified by age, sex, and ethnic background. The two tests were followed by a shoot and move test tailored to tactical athletes to assess reaction times to visual stimuli, using a simulation handgun (Glock 17T FX, GLOCK GmbH, Deutsch-Wagram, Austria) loaded with a 0.4 g plastic projectile (Simunition^®^; General Dynamics Ordnance and Tactical Systems Canada Inc., Repentigny, QC, Canada) and the Fitlight^®^ system (FITLIGHT Sports Corp., Aurora, ON, Canada) with three Fitlight^®^ pods to measure reaction time. The reaction time test was performed twice after a standardized warm-up (7–10 min on a Technogym synchro crosstrainer (Cesena, Italy), followed by functional gymnastics).

Thereafter, physical performance was in sequence assessed by means of the following test battery: (1) Striking power of the leg (knee and lowkick) and arm (elbow and hammer fist) impact power, using a striking power measuring device (PowerKubeTM, Strike Research Limited, Norwich, UK). (2) Handgrip strength on both hands as measured using a hydraulic hand dynamometer (SH5001^®^, Saehan Corporation, Changwon, Republic of Korea) with the participants in the seated position. (3) Upper body strength endurance, assessed by the number of pullups while wearing a 12.5 kg weighted vest from an overhand grip hanging position, and the one-repetition maximum (1 RM) was estimated using the 1 RM calculator as a proxy of muscle strength (https://strengthlevel.com/one-rep-max-calculator, last accessed on 17 December 2025). (4) Fatigue resistance during a loaded cardiopulmonary exercise test (CPET) consisting of uphill treadmill running until exhaustion with a 12.6 kg vest, performed at 8 km/h with the incline increasing by 0.1° every 6 s as described [59]. Pulmonary gas exchange was measured breath-by-breath (MetaMax 3B-R2), heart rate via chest-strap sensor (Polar H10), and systemic and local muscle oxygenation using two near-infrared spectroscopy (NIRS) devices (Moxy, Fortiori Design LLC., Hutchinson, MN, USA) positioned over *m. vastus lateralis* and *m. gastrocnemius*. Pre- and post-test glucose and lactate concentrations were determined enzymatically from 10 µL capillary ear-lobe samples using a photometer (Lactate Photometer Plus DP 110, Diaglobal, Berlin, Germany). Muscle stiffness was evaluated at five standardized sites using triple measurements with a Digital Palpation Device (MyotonPRO, Myoton AS, Tallinn, Estonia). (5) A 20-min break was provided during which participants reported their training habits for each day of the previous week. Self-reported training habits were assessed using questions about the type of training performed (e.g., explosive/maximal strength, strength/hypertrophy, strength-endurance, endurance, coordination, tactical, sport-specific), volume (session duration in minutes) for each day, distinguishing between morning and afternoon sessions to minimize recall bias. Perceived effort for endurance training was classified into three zones: basic zone 1 (light; 60–75% of maximal heart rate or VO_2_max), basic zone 2 (moderate; 75–85%), and zone 3 (high; >85%). Training minutes at each intensity level were summed to calculate total weekly hours per intensity category. Additionally, physical and mental strain and recovery were monitored using a six-item inventory [106]. The questionnaire was normed using large samples of athletes, which means that a score of 0 is the strain and recovery of an average sports athlete, with negative scores representing strain exceeding recovery. (6) Finally, participants completed an all-out deadlift test to assess leg and lower-back strength endurance and to estimate 1RM (using the same equations as for pull-ups). The test was performed with a 20 kg Olympic barbell loaded with 80 kg, following standardized instructions and a video-demonstrated technique briefing. Buccal swabs were collected in a preserving solution (DNA/RNA Shield Collection Tube w/Swab, R1109, Zymo Research, Distributor Lucerna-Chem AG, Lucerne, Switzerland), and kept at a temperature of 4–8 °C for up to two weeks, before being subjected to the typing of gene polymorphisms *COMT* (rs4680) and *ACE* (rs1799752). Samples of around 200 microliters of capillary blood were taken before and immediately after the test battery, spotted and dried on membranes (QIAcard FTA TM Classic, Qiagen, Hombrechtikon, Switzerland), and kept in sealed plastic bags between 4 and 8 °C for the subsequent analysis of the methylation of CpG dinucleotides in the *COMT* and *ACE* promoters. Data were recorded anonymously, using a pseudonymized Study-ID, provided by their employers or the recruiting authority.

### 4.4. Calculation of Fitness Response Variables

Ventilatory thresholds (VTs) 1 and 2, and VO_2_max (or, if no VO_2_ plateau was reached, the peak value, VO_2_peak) were determined by a sport scientist who was highly experienced in CPET using a structured assessment approach based on panels 5 (V-slope), 6, 1, and 9 of Wasserman’s 2012 revised 9-panel-plot [107].

Performance during the treadmill run was calculated as the energetic cost of level running plus the vertical mechanical power required to raise body mass against gravity, accounting for the additional weight carried, essentially as described [108]. Oxygen uptake and biomechanical performance at ventilatory thresholds were used as fitness response variables.

### 4.5. Genotyping

Typing of polymorphisms in the *COMT* (rs4680, G/A) and *ACE* genes (rs1799752, insertion(I)/deletion(D)) was conducted by a melting curve analysis versus validated references in polymerase chain reactions on genomic DNA from the buccal swabs with the exact conditions as described [71]. On the occasion of inconclusive results, reactions were repeated and sequenced to validate the detected genotype by Sanger sequencing using a commercial provider (Microsynth, Balgach, Switzerland).

### 4.6. DNA Methylation

Before the maximal storage duration of 2 months had elapsed, DNA was isolated from the dried capillary blood samples on the membranes. First, the circle holding the biological material was cut into ~2 mm^2^ large pieces with the help of clean scissors in a sterile hood. Subsequently, the DNA was extracted from the minced membranes using a total of 400 microliters of phosphate buffered saline in a 1.5-mL microcentrifuge tube (Biosphere^®^ SafeSeal tube, Sarstedt, Nümbrecht, Germany) by repeated steps of vortexing and centrifugation, and incubations for 1 h at 37 °C followed by 8 h at room temperature (~20 °C). The available supernatant (~200 microliter) was subjected to a silica-gel-membrane-based DNA isolation using the DNeasy blood and tissue kit (Cat No. 69504, Qiagen, Hombrechtikon, Switzerland) and extracted into 150 microliters of sterile, nuclease free H2O (Ultrapure, DNase/RNase Free, Invitrogen, Carlsbad, CA, USA). The relative concentration of isolated DNA was qualitatively assessed from the detection cycle and amplification efficiency of the *ACE* genotype rs1799752 in subsequent PCR reactions, and in instances based on UV spectroscopy using a nanodrop device.

Twenty microliters of the DNA containing solution was subjected to a bisulfite-conversion with the EZ DNA Methylation-Lightning kit (Zymo Research, LucernaChem, Luzern, Switzerland), following the generic protocol with denaturation for 12 min at 98 °C, followed by a reaction in lightning for 60 min at 54 °C, a desulfonation step, and column based purification in a microcentrifuge at room temperature. Half of the final purified material in 10 microliters was subjected to specific PCR reactions to amplify respective promoter regions of the *COMT* and *ACE* genes, with specific oligonucleotides (see Figure 3 for a display of investigated sequences and exemplary analysis), as adapted from [35,36]. The amplification was carried out with KAPA SYBR FAST Mix (KK4603, Kapa Biosystems, ABI Prism, Merck, Buchs, Switzerland) in a magnetic induction cycler (Mic Real-Time PCR System, Bio Molecular Systems; Labgene, Châtel-St-Denis, Switzerland). Reaction products were subjected to Sanger sequencing on a SEQ-ABI02 DNA analyzer (Applied Biosystems, Foster City, CA, USA) by a commercial provider (Microsynth, Balgach, Switzerland) with specific primers, i.e., 5′-GAGTAGGTTGTGGATGGGTTGTA-3′, 5′-ACATTTCTAAACCTTACCCCTCTA-3′ for *COMT* and 5′-TTATGGTTTGGTGAAGAAGT-3′, 5′-AAAAAAACCTCCTCTCTTTAAA-3′ for *ACE*. Chromatograms were first inspected for consistency and visualized using the CutePeaks software (version 0.2.3, https://appimage.github.io/CutePeaks/, accessed on 26 November 2023).

The cytosine methylation in CpG dinucleotides was estimated from the ratios of the signals for protected (due to methylation) and unprotected (i.e., converted) cytosine nucleotides. This was based on a semi-automatic algorithm (macro) in MS-Excel (Microsoft^®^ Excel^®^ for Microsoft 365 MSO (Version 2501 Build 16.0.18429.20132) 64 bit) on csv-files for the sequence reads that were exported from the chromatograms. The “final” promoter methylation was calculated from the average of estimated ratios in the ratio of protected vs. the sum of unprotected + protected cytosine residues. Recurring sequence anomalies at specific nucleotide positions and results were validated in instances in separate digital and analog assessment of the respective signal intensities in the chromatograms.

### 4.7. Quality Control

All study procedures were performed in accordance with internal standard operating procedures. Data acquisition and processing were monitored to ensure adherence to protocol and to verify the quality of execution. Where possible, quality control checks, including internal/external references, enabled the assessment of the precision and accuracy of the deployed techniques at the levels of individual and repeated measurements. Indices of the reliability of the deployed physical and cognitive tests are given in Table S1. The coefficient variation in repeated measurements in different batches for *ACE*/*COMT* promoter methylation was 17.5% ± 6.5% and 13.2% ± 7.6%. The call rate for correct genotyping of *ACE* and *COMT* gene polymorphisms rs1799752 and rs4680 were 99.3% and 95.97%, respectively.

### 4.8. Data Handling

All study data, including data from questionnaires and inventories, were collected and managed in pseudonymized form using REDCap electronic data capture tools (v13.7.3) [109] hosted at the Federal Office of Sport, FOSPO, Magglingen, Switzerland. For the purpose of this publication, relevant data were extracted and curated in MS-Excel. For a list of the handled variables and their abbreviations, we refer to Table S1.

### 4.9. Statistics

Values are summarized as mean ± standard deviation (SD). Retrospective power analysis was carried out for a bivariate normal model using Pearson’s correlation coefficient (α = 0.05) in G*Power (version 3.1.9.7; https://www.psychologie.hhu.de/arbeitsgruppen/allgemeine-psychologie-und-arbeitspsychologie/gpower, accessed on 23 September 2015).

The Hardy–Weinberg equilibrium (HWE) was assessed for the *ACE* (rs1799752, *I/D*) and *COMT* (rs4680, G/A) gene polymorphism using frequentist and Bayesian approaches to address limitations of chi-square tests in a moderate sample. Exact tests were performed with the HardyWeinberg (version 1.7.9) R package [110]. For the Bayesian assessment of HWE, two competing models were compared [111]: a constrained model (M0), assuming HWE with genotype frequencies determined by a single allele-frequency parameter assigned a Beta(1,1) prior, and an unconstrained model (M1), estimating genotype frequencies freely with a Dirichlet(1,1,1) prior. Evidence was quantified using the Bayes factor (BF), defined as the ratio of marginal likelihoods (M0/M1), where BF > 1 supports HWE and BF < 1 indicates deviation. Robustness was tested using Jeffreys-type priors (Beta(0.5,0.5); Dirichlet(0.5,0.5,0.5)).

Associations between (epi)genetic markers and performance variables were examined using Pearson correlations under an additive coding scheme: each *ACE I*-allele and *COMT* rs4680 *G*-allele contributed one unit, with the respectively complementary *D*- and *A*-alleles coded as zero, reflecting the additive positive influence of respective alleles on aerobic metabolism and performance [71]. Promoter methylation values were entered as the corresponding cytosine-methylation ratios. Linear regression analysis was carried out to estimate the in-sample model-fit of a dependent parameter based on the 4 covariates *ACE* genotype, *ACE*-promoter methylation, *COMT* genotype and *COMT*-promoter methylation. Assumption testing followed standard procedures for linear regression, as described by Field (2018) [112]. Multicollinearity was assessed using the Variance Inflation Factor (VIF) and tolerance statistics, independence of residuals using the Durbin–Watson test, and normality of residuals using Q–Q plots and the Shapiro–Wilk test. Pre–post exercise differences were assessed with a repeated-measures analysis of variance. Analyses were carried out using JASP software (version 0.18.3.0, University of Amsterdam, https://jasp-stats.org/download/ (accessed on 9 January 2024)).

Statistical significance was defined as *p* < 0.05, with a false discovery rate (FDR) adjustment applied at *q* < 0.05 to account for multiple testing, essentially as described in [113], and calculated in MS-Excel (Microsoft^®^ Excel^®^). The strength of linear relationships was classified as weak (|r| = 0.1 to 0.3), moderate (|r| = 0.3 to 0.5) or strong correlations (|r| > 0.5). Correlation coefficients were evaluated against the FDR-adjusted 5% threshold across the seven resilience-related dependent variables: perceived strain, cognitive resilience, physical strength, mechanical resistance, metabolic capacity, metabolic recovery, and behavior. Significant relationships were visualized using publicly available Cluster and Treeview software (version 2.11 and 1.60, https://diyhpl.us/~bryan/irc/protocol-online/protocol-cache/EisenSoftware.htm, accessed on 29 March 2025) and Cytoscape software (version 3.10.3, https://cytoscape.org/, accessed on 21 November 2023) following curation of the data in MS-Excel software (Microsoft Corporation (2024), 365 subscription).

## 5. Conclusions

In the studied cohort of Swiss tactical athletes, *COMT* and *ACE* exhibit distinct (epi)genetic associations with resilience-related cognitive and physiological traits. Promoter methylation of *COMT* correlates with cognitive performance under stress and oxygen utilization metrics, whereas *ACE*-promoter methylation is associated with strength–endurance and muscle oxygenation. Antidromic patterns between *COMT* genotype and methylation suggest complex regulatory dynamics. Observed methylation levels appear sensitive to recent training exposure and perceived strain, potentially reflecting adaptive, environment-responsive signaling.

While these (epi)genetic factors contribute modestly to interindividual variability in performance, these data support a biologically plausible neurovascular link involving *ACE*- and *COMT*-gene activity-regulated catecholaminergic and angiotensin pathways. The findings align with the hypothesis that *ACE*- and *COMT*-gene-related profiles may inform personalized conditioning strategies; however, causal inference remains limited due to the observational nature and cohort size of this study. To substantiate the eventual prognostic robustness of identified relationships, exploring site-specific methylation effects and elucidating the underlying (regulatory) mechanisms, larger, longitudinal studies in genetically homogeneous populations is warranted.

## Figures and Tables

**Table 1:** Cohort characteristics.

Variable	Unit	Mean	SD	Min–Max
**Anthropometry**				
Age	Years	24.56	5.61	[18.00–38.00]
Weight	kg	78.40	9.48	[59.55–95.60]
Height	cm	180.78	7.03	[167.60–198.50]
BMI	kg m^−2^	23.96	2.34	[19.00–29.20]
**Aerobic performance**				
VO_2_max	L O_2_ min^−1^	4.38	0.50	[3.00–5.38]
VO_2_max relative	ml O_2_ min^−1^ kg^−1^	56.66	5.97	[43.00–72.00]
P_VO_2_max	Watt	518.7	66.2	[370.0–681.5]
maxP_end	Watt	533.8	67.2	[375.1–705.8]
**Strength**				
handgrip_r	kg	61.92	9.30	[44.00–85.00]
handgrip_l	kg	59.61	9.21	[37.00–78.00]
1rm pullup mass	kg	112.85	17.31	[79.64–152.8]
pullup_rep	Repetitions	8.03	3.69	[2–15]
1rm deadlift mass	kg	168.68	61.80	[92.92–436.7]
deadlift_rep	Repetitions	22.97	17.81	[2–101]
P_hammerf	Watt	24,383.0	7568.3	[11,574–43,690]
P_elbow	Watt	21,655.8	7035.3	[7785–43,512]
P_knee	Watt	22,781.6	8028.4	[12,888–61,417]
P_lowk	Watt	41,855.5	12,318.3	[3453–73,321]
**Cognitive performance**				
STROOP_R_if	Seconds	0.78	0.13	[0.52–1.16]
STROOP_W_if	Seconds	0.65	0.08	[0.47–0.84]
STROOP_pr_R	Percentile rank	46.44	28.35	[0.51–96.92]
STROOP_pr_W	Percentile rank	62.95	29.97	[3.33–100.0]
DT_rt	Seconds	0.66	0.06	[0.54–0.81]
DT_pr_rt	Percentile rank	58.72	29.14	[2.92–99.42]
DTpr_r	Percentile rank	55.05	32.57	[2.33–100.0]
DTpr_w	Percentile rank	44.53	30.33	[1.90–97.96]
DTpr_s	Percentile rank	52.73	28.63	[1.31–97.81]
**Shooting performance**				
shoot_rt	Seconds	2.29	0.24	[1.80–2.60]
shot_miss	Number	2.52	2.09	[0–5]
Training behavior *				
training hours	Hours week^−1^	7.27	3.19	[0–14.25]
Tpc_strength	%	50.10	20.50	[5.00–100.0]
Tpc_endurance	%	40.79	17.85	[0.00–85.00]
Tpc_tactical	%	9.11	13.2	[0.00–68.00]
Tpc_strengthmax	%	6.87	7.99	[0.00–34.00]
Tpc_hypertr	%	23.83	20.43	[0.00–85.00]
Tpc_strengthendur	%	20.38	18.42	[0.00–97.00]
Tpc_ga1	%	19.22	14.07	[0.00–55.00]
Tpc_ga2	%	10.25	10.11	[0.00–35.00]
**Strain** **and** recovery *				
bemi_gess	Score Points	25.36	30.33	[−70.0–88.0]
bemi_erhs	Score Points	13.24	19.03	[−42.0–61.0]
bemi_beans	Score Points	−12.12	16.65	[−33.0–49.0]

Data represent anthropometric, cognitive, aerobic, strength, and shooting performance, as well as training behavior and strain and recovery of the 61 tactical athletes studied. * in a (typical) week preceding the test battery. For further explanation, we refer to the list of abbreviations and Table S1.

## Data Availability

The data presented in this study are available upon request from the corresponding author due to restrictions related to the protected nature of the subjects studied.

## Supplementary Materials

The following supporting information can be downloaded at: https://www.mdpi.com/article/10.3390/ijms27031340/s1. Refs. [114,115,116,117,118,119,120,121,122,123,124,125,126,127,128,129,130,131,132,133,134] are cited in Supplementary Materials file.

## Abbreviations

The following abbreviations are used in this manuscript. For further descriptions of abbreviations, the reader is referred to Table S1.

ACE_gt	ACE genotype
ACE_pmet	Mean percentage methylation of the ACE promoter
bemi_gess	Overall physical and mental strain and recovery score in sports (Ger.: Gesamtscore [gess])
bemi_erhs	Recovery score in sports (Ger.: Erholungsscore [erhs])
bemi_beans	Strain score in sports (Ger.: Beanspruchungsscore [beans])
Bm	Body mass
BMI	Body mass index
COMT_gt	COMT genotype
COMT_pmet	Mean percentage methylation of the COMT promoter
DT_rt	Reaction time in the determination test
DTpr_r	Percentage rank of correct answers in the determination test
DTpr_s	Percentage rank of skipped answers in the determination test
DTpr_w	Percentage rank of wrong answers in the determination test
endurance	Endurance-specific physical training
handgrip_r	Maximal isometric kg-force of the right palm
handgrip_l	Maximal isometric kg-force of the left palm
height	Body height
maxP_end	Maximal performance during running (weight of body mass + vest)
P_VO_2_max	Performance at maximal oxygen uptake during running (weight of body mass + vest)
Q	Cardiac output during running exercise
Q_D	Changes in cardiac output during recovery
RER	Respiration exchange rate during running exercise
SmO_2_	Muscle oxygen saturation
SmO_2__GM	Oxygen saturation in gastrocnemius muscle
SmO_2__VL	Oxygen saturation in vastus lateralis muscle
Strength	Strength specific training
STROOP_pr_rt	Percentage rank in the reaction time (STROOP)
STROOP_R_if	Reading interference
STROOP_W_if	Writing interference
STROOP_pr_R	Percentage rank in reading
STROOP_pr_W	Percentage rank in writing
Tactical	Tactic specific training
tHb	Hemoglobin concentration
tHb_VL	Hemoglobin concentration in vastus lateralis muscle
VO_2_	oxygen uptake
VO_2__D	changes in oxygen uptake during recovery
VO_2_max	Maximal oxygen uptake
VT	Ventilatory threshold
