# Supplementary material for: COMT and ACE (Epi)genetic Variation Is Associated with Cognitive and Metabolic Resilience in Swiss Tactical Athletes
Source: Int J Mol Sci. 2026 Jan 29;27(3):1340. doi: 10.3390/ijms27031340 (PMC12898589; doi:10.3390/ijms27031340)
Supplement: Supplementary file 1 [file ijms-27-01340-s001.zip › Table S1.pdf]

Table S1: *Grouping of assessed variables to functional aspects (traits).*

variable (abbreviation)	test	unit	functional aspect	organic system	variable (explanation)	Reliability r2 (CV)	reference
bemi_gess	six-item inventory	score	prerceived strain	system	overall strain score in sports		[106]
bemi_psy	six-item inventory	score	prerceived strain	system	mental strain overall strain score in sports		[106]
bemi_phys	six-item inventory	score	prerceived strain	system	physical strain score in sports		[106]
bemi_erhs	six-item inventory	score	prerceived strain	system	recovery score in sports		[106]
bemi_beans	six-item inventory	score	prerceived strain	system	average score in sports		[106]
shoot rt	Fitlight® system based shooting	sec	cognitive performance	brain & actuators	reaction time 1st trial		[97]
shoot_miss	Fitlight® system based shooting	number	cognitive performance	brain & actuators	misses in 1st trial		[97]
STROOP_pr_R	color-word interference tendency test	percentile rank	cognitive performance	brain	percentage rank in naming read text	0.85-0.99	[41, 114, 115]
STROOP_pr_W	color-word interference tendency test	percentile rank	cognitive performance	brain	percentage rank in naming recognizing written text	0.85-0.99	[41, 114, 115]
shoot_rt_D	Fitlight® system based shooting	sec	cognitive resilience	brain & actuators	difference reaction time between trial 1 and 2		[97]
shoot_miss_D	Fitlight® system based shooting	number	cognitive resilience	brain & actuators	difference in misses between trial 1 and 2		[97]
STROOP_R_if	color-word interference tendency test	sec	cognitive resilience	brain	read interference	0.85-0.99	[41, 114, 115]
STROOP_W_if	color-word interference tendency test	sec	cognitive resilience	brain	write interference	0.85-0.99	[41, 114, 115]
DT_rt	determination test for reactive stress tolerance	sec	cognitive performance	brain	median reaction time	0.98-0.99	[41, 116]
DT_pr_rt	determination test for reactive stress tolerance	percentile rank	cognitive performance	brain	percentage rank of median reaction time	0.98-0.99	[41,116]

DT_pr_r	determination test for reactive stress tolerance	percentile rank	cognitive resilience	brain	right answer	0.98-0.99	[41, 116]
DT_pr_w	determination test for reactive stress tolerance	percentile rank	cognitive resilience	brain	wrong answer	0.98-0.99	[41, 116]
DT_pr_s	determination test for reactive stress tolerance	percentile rank	cognitive resilience	brain	skipped answer	0.98-0.99	[41, 116]
hand_r	maximal isometric force of hand palm	kg	physical performance (strength)	muscle	right hand	0.939	[118, 119]
hand_l	maximal isometric force of hand palm	kg	physical performance (strength)	muscle	left hand	0.939	[118, 119]
P_elbow	impact power	Watt	physical performance (impulse)	muscle	upper body power of elbow strike	(3.6%)	[97]
P_hammerf	impact power	Watt	physical performance (impulse)	muscle	upper body power of hammer fist strike	(3.6%)	[97]
P_knee	impact power	Watt	physical performance (impulse)	muscle	lower body power of knee strike	(3.6%)	[97]
P_lowk	impact power	Watt	physical performance (impulse)	muscle	lower body power of low kick	(3.6%)	[97]
1RM_pullup	power	kg	physical performance (strength)	muscle	One repetition maxima of weight to be pulled up (calculated)	0.92	[97, 120]
1RM_deadlift	power	kg	physical performance (strength)	musculoskeletal system	One repetition maxima of weight to be lifted (calculated)	0.92	[97, 120]
pullups_rep	pull ups with weight vest	repetitions	physical performance (strength-endurance)	musculoskeletal system	repetition number		[97]
deadlifts_rep	deadlift	repetitions	physical performance (strength-endurance)	musculoskeletal system	repetition number		[97]
P_VT1_V	loaded uphill running into exhaustion	Watt	physical performance (strength endurance)	musculoskeletal system	power at the 1st ventilatory threshold	0.97	[97, 98, 121]
P_VT2_V	loaded uphill running into exhaustion	Watt	physical performance (strength endurance)	musculoskeletal & cardiovascular system	power at the 2nd ventilatory threshold	0.97	[97, 98, 121]

P_VO2max_V	loaded uphill running into exhaustion	Watt	physical performance (strength endurance)	musculoskeletal & cardiovascular system	power at maximal O2 uptake	0.97	[42, 98, 122, 123]
maxP_end_V	loaded uphill running into exhaustion		physical performance (strength endurance)	musculoskeletal & cardiovascular system	maximal power	0.97	[42, 98, 122, 123]
VO2_start	loaded uphill running into exhaustion	L O2 min-1	metabolic capacity	cardiorespiratory system	O2 uptake at baseline	0.98 (3%)	[98, 107, 124-126]
VO2_vt1	loaded uphill running into exhaustion	L O2 min-1	metabolic capacity	cardiorespiratory system	O2 uptake at the frist ventilatory threshold	0.98 (3%)	[98, 107, 124-125]
VO2_vt2	loaded uphill running into exhaustion	L O2 min-1	metabolic capacity	cardiorespiratory system	O2 uptake at the frist 2nd threshold	0.98 (3%)	[98, 107, 124-125]
VO2max	loaded uphill running into exhaustion	L O2 min-1	metabolic capacity	cardiorespiratory system	O2 uptake at maximal O2 uptake	0.98 (3%)	[98, 107, 124-125]
Q_start	loaded uphill running into exhaustion	L O2 min-1	metabolic capacity	cardiorespiratory system	cardiac output at baseline	0.82	[107, 124, 125, 127]
Q_vt1	loaded uphill running into exhaustion	L O2 min-1	metabolic capacity	cardiorespiratory system	cardiac output at the frist ventilatory threshold	0.82	[107, 124, 125, 127]
Q_vt2	loaded uphill running into exhaustion	L O2 min-1	metabolic capacity	cardiorespiratory system	cardiac output at the frist 2nd threshold		[107, 124, 125]
Q_vo2max	loaded uphill running into exhaustion	L O2 min-1	metabolic capacity	cardiorespiratory system	cardiac output at maximal O2 uptake		[107, 124, 125]
Q_max	loaded uphill running into exhaustion	L O2 min-1	metabolic capacity	cardiorespiratory system	maximal cardiac output		[107, 124, 125]
tHb_VAS_start	loaded uphill running into exhaustion	g dL-1	metabolic capacity	muscle cardiovasculature	hemoglobin conc in vastus lateralis muscle at rest	0.90 (10%)	[64, 128, 129]
tHb_VAS_vt1	loaded uphill running into exhaustion	g dL-1	metabolic capacity	muscle cardiovasculature	hemoglobin conc in vastus lateralis 1st ventilatory threshold	0.90 (10%)	[64, 128, 129]
tHb_VAS_vt2	loaded uphill running into exhaustion	g dL-1	metabolic capacity	muscle cardiovasculature	hemoglobin content in vastus lateralis 2nd threshold		[64, 128]
tHb_VAS_vo2max	loaded uphill running into exhaustion	g dL-1	metabolic capacity	muscle cardiovasculature	hemoglobin content in vastus lateralis at maximal O2 uptake		[64, 128]

tHb_VAS_stop	loaded uphill running into exhaustion	g dL-1	metabolic capacity	muscle cardiovasculature	hemoglobin content in vastus lateralis at max performance		[64, 128]
RER_start	loaded uphill running into exhaustion	without unity	metabolic capacity	muscle & cardiorespiratory system	respiration exchange rate at rest	0.97 (3%)	[107, 124-126, 127]
RER_vt1	loaded uphill running into exhaustion	without unity	metabolic capacity	muscle & cardiorespiratory system	respiration exchange rate at the 1st ventilatory threshold	0.97 (3%)	[107, 124-126, 127]
RER_vt2	loaded uphill running into exhaustion	without unity	metabolic capacity	muscle & cardiorespiratory system	respiration exchange rate at the 1st 2nd threshold	0.97 (3%)	[107, 124-126, 127]
RER_vo2max	loaded uphill running into exhaustion	without unity	metabolic capacity	muscle & cardiorespiratory system	respiration exchange rate at maximal O2 uptake	0.97 (3%)	[107, 124-126, 127]
RER_max	loaded uphill running into exhaustion	without unity	metabolic capacity	muscle & cardiorespiratory system	respiration exchange rate at maximal performance	0.97 (3%)	[107, 124-126, 127]
SmO2_VAS_start	loaded uphill running into exhaustion	%	metabolic capacity	muscle	O2 saturation in vastus lateralis muscle at rest	~0.90 (4.6%)	[64, 128, 130, 131]
SmO2_VAS_vt1	loaded uphill running into exhaustion	%	metabolic capacity	muscle	O2 saturation in vastus lateralis at 1st ventilatory threshold	~0.84 (4.6%)	[64, 128, 130, 131]
SmO2_VAS_vt2	loaded uphill running into exhaustion	%	metabolic capacity	muscle	O2 saturation in vastus lateralis at 2nd threshold	~0.92	[64, 128, 130]
SmO2_VAS_vo2max	loaded uphill running into exhaustion	%	metabolic capacity	muscle	O2 saturation in vastus lateralis at maximal O2 uptake	~0.93	[64, 128, 130]
SmO2_VAS_stop	loaded uphill running into exhaustion	%	metabolic capacity	muscle	O2 saturation in vastus lateralis at maximal performance	~0.90	[64, 128, 130]
SmO2_GAS_start	loaded uphill running into exhaustion	%	metabolic capacity	muscle	O2 saturation in gastrocnemius muscle at rest	~0.74	[64, 128, 130]
SmO2_GAS_vt1	loaded uphill running into exhaustion	%	metabolic capacity	muscle	O2 saturation in gastrocnemius at 1st ventilatory threshold	~0.74	[64, 128, 130, 132]
SmO2_GAS_vt2	loaded uphill running into exhaustion	%	metabolic capacity	muscle	O2 saturation in gastrocnemius at 2nd threshold	~0.74-0.92	[64, 128, 130]

SmO2_GAS_vo2max	loaded uphill running into exhaustion	%	metabolic capacity	muscle	O2 saturation in gastrocnemius at maximal O2 uptake	~0.74-0.93	[64, 120, 130]
SmO2_GAS_stop	loaded uphill running into exhaustion	%	metabolic capacity	muscle	O2 saturation in gastrocnemius at maximal performance	~0.74-0.90	[64, 128, 130]
Glu_pre	loaded uphill running into exhaustion	mmol	metabolic capacity	muscle and hepatic system	blood serum glucose prior to running exercise	(0.5%)	[64, 117, 128]
Glu_post	loaded uphill running into exhaustion	mmol	metabolic capacity	muscle and hepatic system	change in blood serum glucose 5 minutes after running exercise	(0.5%)	[64, 117, 128]
Lac_post	loaded uphill running into exhaustion	mmol	metabolic recovery	muscle	change in blood lactate 5 minutes after running exercise	(0.5%)	[64, 117, 128]
VO2_D	loaded uphill running into exhaustion	L O2 min-1	metabolic resilience	cardiorespiratory system	changes in O2 uptake during recovery	0.97 (4%)	[64, 126, 128]
Q_D	loaded uphill running into exhaustion	L O2 min-1	metabolic recovery	cardiorespiratory system	changes in cardiac output during recovery	~0.82	[107, 124, 125, 127]
tHb_D	loaded uphill running into exhaustion	mg L-1	metabolic recovery	muscle cardiovasculature	change hemoglobin content in vastus lateralis during recovery		[64, 128]
SmO2_VAS_D	loaded uphill running into exhaustion	%	metabolic recovery	muscle	O2 saturation change in vastus lateralis during recovery		[64, 128]
SmO2_GAS_D	loaded uphill running into exhaustion	%	metabolic recovery	muscle	O2 saturation change in gastrocnemius during recovery		[64, 128]
Glu_D	loaded uphill running into exhaustion	mmol	metabolic recovery	muscle and hepatic system	change in blood serum glucose during recovery	(0.5%)	[64, 117, 128]
Lac_D	loaded uphill running into exhaustion	mmol	metabolic recovery	muscle	change in blood lactate during recovery	(0.5%)	[64, 117, 128]
stiff_PT_D	loaded uphill running into exhaustion	N/m	mechanical resilience	muscle	change of plantaris stiffness during recovery	0.92	[133, 134]
stiff_VL_D	loaded uphill running into exhaustion	N/m	mechanical resilience	muscle	change of vastus lateralis stiffness during recovery	0.92	[133, 134]
stiff_RF_D	loaded uphill running into exhaustion	N/m	mechanical resilience	muscle	change of rectus femoris stiffness during recovery	0.92	[133, 134]
stiff_MS_D	loaded uphill running into exhaustion	N/m	mechanical resilience	muscle	change of soleus muscle stiffness during recovery		[134]

stiff_AT_D	loaded uphill running into exhaustion	N/m	mechanical resilience	muscle	change of Achilles tendon stiffness during recovery	0.92	[133, 134]
VO2_rec	loaded uphill running into exhaustion	L O2 min-1	metabolic resilience	cardiorespiratory system	oxygen uptake 2 minutes after running exercise		[107, 124, 125]
Tpc_end	questionnaire	%	behavior	cardiorespiratory system	conducted percentage of endurance training		
Tpc_GA1	questionnaire	%	behavior	cardiorespiratory system	conducted percentage of training in basic endurance zone 1		[97]
Tpc_GA2	questionnaire	%	behavior	cardiorespiratory system	conducted percentage of training in basic endurance zone 2		[97]
Tpc_hypert	questionnaire	%	behavior	muscle	conducted percentage of hypertrophy training		[97]
Tpc_koopr	questionnaire	%	behavior	brain	conducted percentage of tactical training		[97]
Tpc_str_endu	questionnaire	%	behavior	muscle	conducted percentage of strength-endurance training		[97]
Tpc_streng_max	questionnaire	%	behavior	muscle	conducted percentage of strength training at maximal load		[97]
Tpc_tac	questionnaire	%	behavior	brain	conducted percentage of training tactical training		[97]

List of quantified variables, the deployed test, and the respectively assessed functional aspects, type and aspect of function, mainly reflected organic system, explained variable, and the typical reliability of the test as reported in here or elsewhere.
