# Supplementary material for: COMT and ACE (Epi)genetic Variation Is Associated with Cognitive and Metabolic Resilience in Swiss Tactical Athletes
Source: Int J Mol Sci. 2026 Jan 29;27(3):1340. doi: 10.3390/ijms27031340 (PMC12898589; doi:10.3390/ijms27031340)
Supplement: Supplementary file 1 [file ijms-27-01340-s001.zip › Table S2.pdf]

Table S2: *Metabolic and mechanical aspects of resilience.*

variable	time point	mean	SD	p-value	η^2	n
stiffness PT	pre	735.0	235.8			
[N/m]	post	704.7	220.7			
	% change	-4.1%		0.017	0.102	49
stiffness VL	pre	255.8	34.0			
[N/m]	post	244.3	40.4			
	% change	-4.5%		0.006	0.133	57
stiffness RF	pre	266.1	33.2			
[N/m]	post	265.1	32.2			
	% change	-0.4%		0.690	0.003	55
stiffness MS	pre	274.0	48.3			
[N/m]	post	258.2	44.4			
	% change	-5.7%		<0.001	0.256	55
stiffness AT	pre	839.9	141.6			
[N/m]	post	848.5	172.7			
	% change	1.0%		0.663	0.004	55
SmO2 VL	pre	64.69	12.47			
[%]	post	11.71	8.95			
	% change	-81.9%		<0.001	0.936	57
	% recovered	513.1%				
SmO2 GM	pre	66.98	10.74			
[%]	post	13.58	6.84			
	% change	-79.7%		<0.001	0.937	51
	% recovered	454.7%				
tHb VL	pre	12.5	0.4			
mg dL-1	post	12.6	0.4			
	% change	0.8%		<0.001	0.258	49
	% recovered	-0.1%				
blood	pre	5.60	0.68			
glucose	post	6.75	1.03			
[mM]	% change	20.6%		<0.001	0.545	60
	% recovered	3.7%				

Statistical sizes of running exercise effects on mechanical and metabolic properties. Values were sampled at baseline prior to running and immediately after exhaustion. Repeated measures ANOVA with post-hoc test of Holm. Abbreviations: AT, Achilles tendon; GM, m. gastrocnemius medialis; MS, m. soleus; RF, m. rectus femoris; SmO2, muscle oxygen saturation; PT, m. plantaris; tHb, muscle hemoglobin content; VL, m. vastus lateralis.
