# Supplementary material for: COMT and ACE (Epi)genetic Variation Is Associated with Cognitive and Metabolic Resilience in Swiss Tactical Athletes
Source: Int J Mol Sci. 2026 Jan 29;27(3):1340. doi: 10.3390/ijms27031340 (PMC12898589; doi:10.3390/ijms27031340)
Supplement: Supplementary file 1 [file ijms-27-01340-s001.zip › Table S3.pdf]

Table S3: *Retrospective power analysis on identified linear relationships*

(epi)genetic factor	variable	r	p	power
ACE_gt	RER_max	-0.280	0.008	0.851
ACE_gt	Tpc_GA1	0.353	<0.001	0.964
ACE_gt	Tpc_streng_max	-0.314	0.003	0.917
ACE_gt	Tpc_tac	0.226	0.033	0.692
ACE_pmet	P_elbow	-0.209	0.026	0.627
ACE_pmet	pullups_Rep	0.221	0.038	0.670
ACE_pmet	SmO2_GAS_VT1	-0.243	0.030	0.708
ACE_pmet	SmO2_VAS_stop	-0.238	0.027	0.719
ACE_pmet	SmO2_VAS_VT2	-0.272	0.014	0.799
ACE_pmet	stiff_PT_D	-0.264	0.016	0.786
ACE_pmet	stiff_VL_D	-0.260	0.018	0.774
ACE_pmet	Tpc_GA2	-0.215	0.043	0.653
ACE_pmet	Lac_post	0.230	0.030	0.706
COMT_gt	DT_pr_rt	0.356	0.001	0.952
COMT_gt	DT_rt	-0.323	0.003	0.909
COMT_gt	Q_Start	0.267	0.012	0.972
COMT_gt	RER_max	-0.285	0.007	0.862
COMT_gt	RER_start	-0.260	0.015	0.791
COMT_gt	shoot rt	-0.370	<0.001	0.969
COMT_gt	SmO2_VAS_VT1	-0.293	0.006	0.869
COMT_gt	STROOP_W_if	-0.286	0.010	0.830
COMT_gt	VO2_start	0.281	0.008	0.846
COMT_pmet	bemi_beans	-0.456	0.003	0.918
COMT_pmet	bemi_gess	0.412	0.008	0.855
COMT_pmet	Glu_pre	0.353	0.026	0.737
COMT_pmet	Glu_post	0.406	0.011	0.828
COMT_pmet	DT_pr_rt	-0.344	0.034	0.695
COMT_pmet	DT_rt	0.331	0.042	0.663
COMT_pmet	DTpr_w	-0.592	<0.001	0.992
COMT_pmet	Q_D	0.405	0.012	0.826
COMT_pmet	Q_max	0.331	0.037	0.684
COMT_pmet	Q_VO2max	0.329	0.038	0.679
COMT_pmet	RER_VT2	-0.325	0.046	0.649
COMT_pmet	stiff_VL_D	-0.352	0.026	0.734
COMT_pmet	STROOP_W_if	0.324	0.047	0.646
COMT_pmet	tHb_VAS_start	0.580	<0.001	0.990
COMT_pmet	tHb_VAS_VO2max	0.505	<0.001	0.963
COMT_pmet	tHb_VAS_VT1	0.434	0.006	0.875
COMT_pmet	tHb_VAS_VT2	0.451	0.006	0.899
COMT_pmet	Tpc_end	0.440	0.005	0.898
COMT_pmet	Tpc_GA1	0.548	<0.001	0.993
COMT_pmet	Tpc_tac	-0.373	0.018	0.761
COMT_pmet	VO2_D	0.401	0.013	0.818
COMT_pmet	VO2max	0.332	0.036	0.686

Statistical sizes, i.e. r- and p-values and retrospective power, for the identified linear relationships as calculated based on Pearson's Moment correlations and passing a 5% false-discovery rate adjustment for the sixty-one male Swiss tactical athletes. Abbreviations: ACE_pmet, promoter methylation of the

ACE gene; ACE_gt, *ACE* genotype; COMT_pmet, promoter methylation of the *COMT* gene; COMT_gt, *COMT* genotype. For further abbreviations consult table S1.
