# Supplementary material for: COMT and ACE (Epi)genetic Variation Is Associated with Cognitive and Metabolic Resilience in Swiss Tactical Athletes
Source: Int J Mol Sci. 2026 Jan 29;27(3):1340. doi: 10.3390/ijms27031340 (PMC12898589; doi:10.3390/ijms27031340)
Supplement: Supplementary file 1 [file ijms-27-01340-s001.zip › Figure S1.pptx]

## Slide 1
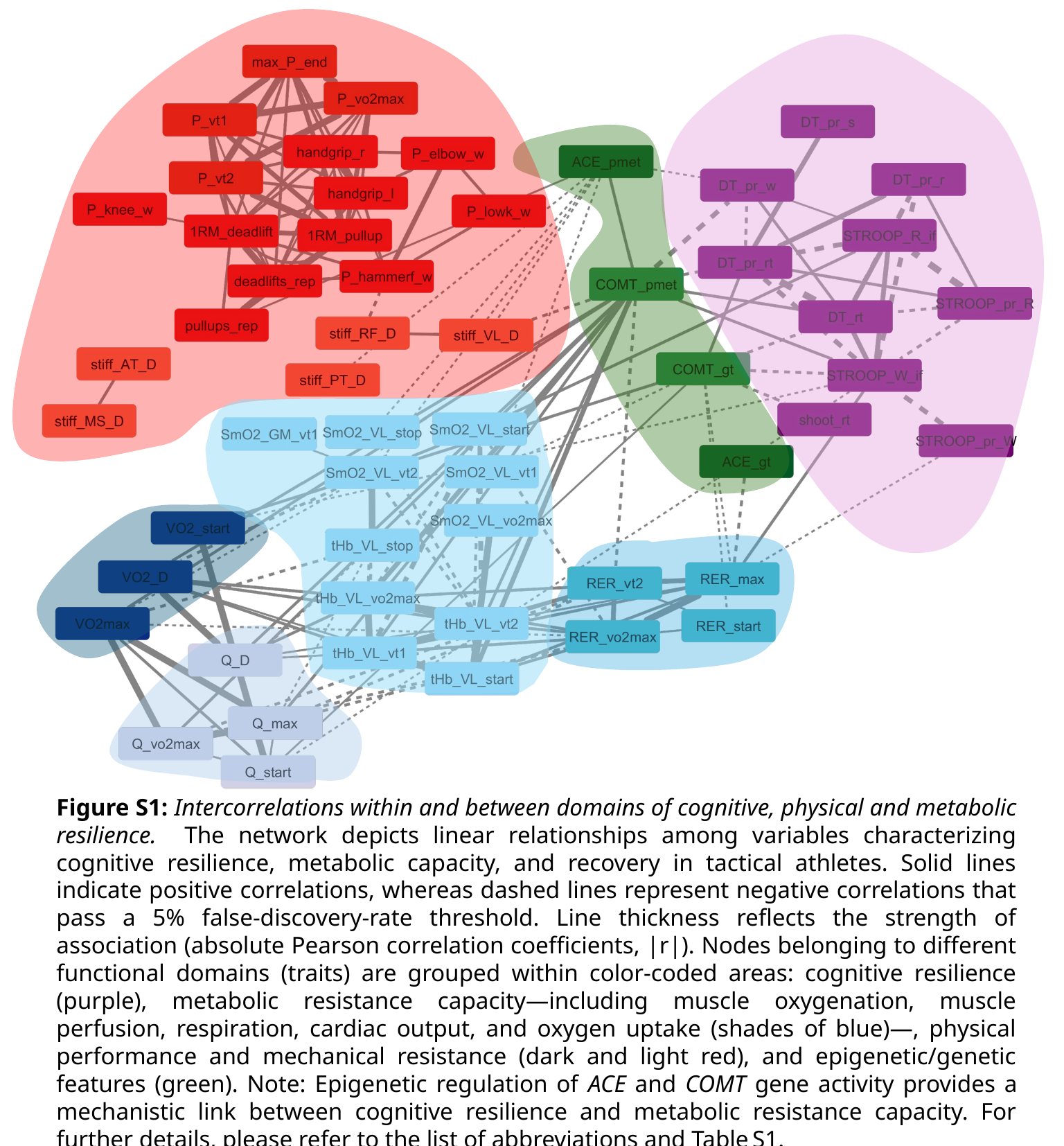

Figure S1: Intercorrelations within and between domains of cognitive, physical and metabolic resilience. The network depicts linear relationships among variables characterizing cognitive resilience, metabolic capacity, and recovery in tactical athletes. Solid lines indicate positive correlations, whereas dashed lines represent negative correlations that pass a 5% false‑discovery‑rate threshold. Line thickness reflects the strength of association (absolute Pearson correlation coefficients, |r|). Nodes belonging to different functional domains (traits) are grouped within color‑coded areas: cognitive resilience (purple), metabolic resistance capacity—including muscle oxygenation, muscle perfusion, respiration, cardiac output, and oxygen uptake (shades of blue)—, physical performance and mechanical resistance (dark and light red), and epigenetic/genetic features (green). Note: Epigenetic regulation of ACE and COMT gene activity provides a mechanistic link between cognitive resilience and metabolic resistance capacity. For further details, please refer to the list of abbreviations and Table S1.
