# Supplementary material for: COMT and ACE (Epi)genetic Variation Is Associated with Cognitive and Metabolic Resilience in Swiss Tactical Athletes
Source: Int J Mol Sci. 2026 Jan 29;27(3):1340. doi: 10.3390/ijms27031340 (PMC12898589; doi:10.3390/ijms27031340)
Supplement: Supplementary file 1 [file ijms-27-01340-s001.zip › Figure S2.pptx]

## Slide 1
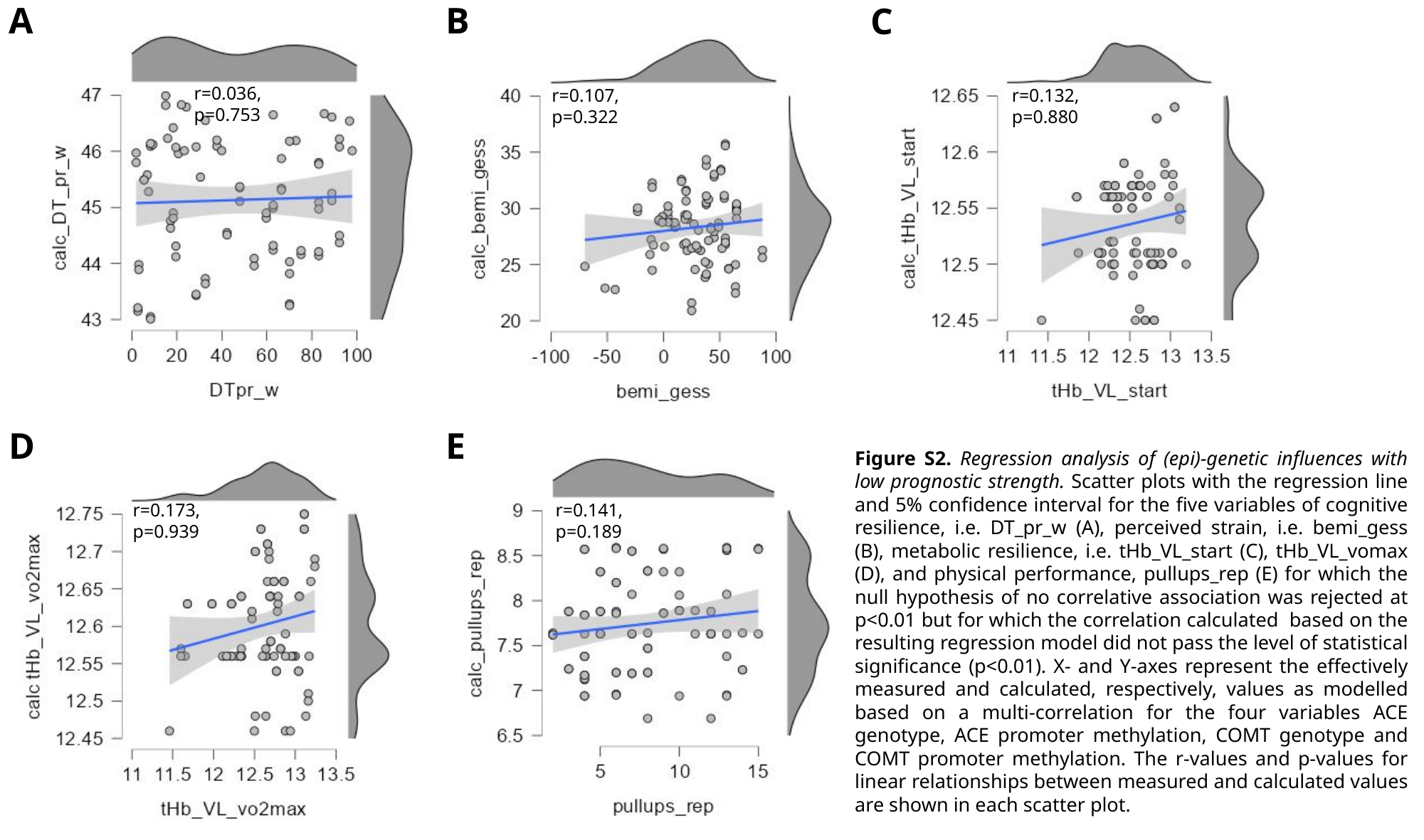

A
B
C
r=0.036, p=0.753
r=0.107, p=0.322
r=0.132, p=0.880
D
E
Figure S2. Regression analysis of (epi)-genetic influences with low prognostic strength. Scatter plots with the regression line and 5% confidence interval for the five variables of cognitive resilience, i.e. DT_pr_w (A), perceived strain, i.e. bemi_gess (B), metabolic resilience, i.e. tHb_VL_start (C), tHb_VL_vomax (D), and physical performance, pullups_rep (E) for which the null hypothesis of no correlative association was rejected at p<0.01 but for which the correlation calculated based on the resulting regression model did not pass the level of statistical significance (p<0.01). X- and Y-axes represent the effectively measured and calculated, respectively, values as modelled based on a multi-correlation for the four variables ACE genotype, ACE promoter methylation, COMT genotype and COMT promoter methylation. The r-values and p-values for linear relationships between measured and calculated values are shown in each scatter plot.
r=0.141, p=0.189
r=0.173, p=0.939
